# Using RET-He and Delta-He in the Sysmex XN-1000V Analyzer to Differentiate Between Chronic Hemorrhagic and Chronic Inflammatory Anemia in Small Animals

**DOI:** 10.3390/ani14223215

**Published:** 2024-11-09

**Authors:** Alejandro Perez-Ecija, Julio Fernandez-Castañer, Carmen Martinez, Francisco J. Mendoza

**Affiliations:** 1 Department of Animal Medicine and Surgery, Campus Rabanales, University of Cordoba, Road Madrid-Cadiz km 396, 14104 Cordoba, Spainfjmendoza@uco.es (F.J.M.); 2 Veterinary Teaching Hospital, Campus Rabanales, University of Cordoba, Road Madrid-Cadiz km 396, 14104 Cordoba, Spain

**Keywords:** anemia, canine, Delta-He, feline, hematology, RET-He

## Abstract

Chronic hemorrhagic anemia (CHA) and anemia of chronic inflammation (ACI) are difficult to differentiate in small animals using hematology. Advanced hematological parameters (RET-He, Delta-He and %Hypo-He) are used in humans to discriminate between types of non-regenerative anemia. Whether they could be useful in the diagnosis of CHA and ACI in small animals is unknown. We evaluated these parameters in the Sysmex XN-1000V analyzer in a population of non-anemic and anemic dogs and cats. Delta-He was significantly different between dogs with CHA and ACI. Moreover, Delta-He and RET-He were different between healthy and non-anemic dogs with inflammation. Neither of these two statements was true for cats. We also report the reference ranges for these parameters using the Sysmex XN-1000V. Although additional clinical and laboratory information should always be considered, the measurement of these parameters using this analyzer can help clinicians to classify type of anemia.

## Simple Summary

Anemia is a common problem in small animals. Veterinary clinicians need to identify the underlying cause of the anemia in their patients for correct treatment and monitoring. The Sysmex XN-1000V analyzer provides advanced parameters (RET-He, Delta-He, %Hypo-He) used in human medicine for classifying anemias. Our results show that the combination of RET-He and Delta-He can help clinicians to differentiate anemias caused by chronic bleeding from those secondary to chronic inflammation. This new approach may facilitate the diagnosis and treatment of these types of anemia in dogs and cats.

## 1. Introduction

Anemia is a common condition in small animals, with a complex differential diagnosis that usually requires a thorough clinical evaluation and multiple laboratory determinations [1,2]. Non-regenerative anemia in veterinary patients can be linked to bone marrow pathologies, renal failure, and primary (absolute) and secondary (functional) iron deficiency, among other causes [3].

In small animals with balanced commercial diets, chronic hemorrhagic anemia (CHA) (gastrointestinal ulceration, thrombocytopenia, parasitosis, etc.) is the main cause of absolute iron deficiency [4,5]. On the other hand, functional iron deficiency is most commonly related to chronic inflammatory diseases, infections, and malignancies, and is usually called anemia of chronic inflammation (ACI) [6,7]. Although the complete range of biochemical processes responsible for ACI is still not totally characterized, hepcidin appears to have a central role [4,8,9]. This peptide, produced by hepatocytes in response to interleukin-6 and other pro-inflammatory mediators, reduces enteric iron absorption and promotes its sequestration in macrophages, causing low levels of available iron for hematopoiesis [7,10].

While other causes of non-regenerative anemia are usually easy to recognize using laboratory results (pancytopenia in myelopathic anemia or azotemia in nephropathic anemia), the laboratory distinction between CHA and ACI is challenging. Recognizing these two types of anemia is clinically important due to their dissimilar prognosis and treatment [11]. Conventional erythrocytic indices lack sensitivity to differentiate between these conditions and, although CHA is traditionally linked to microcytic and hypochromic anemia and ACI to normocytic and normochromic anemia, overlap can be seen in some patients [4,12]. Moreover, changes in the mean corpuscular volume (MCV) or the mean corpuscular hemoglobin concentration (MCHC) must be present for a long period of time in order to affect most erythrocytes and be detectable [4,10,13,14]. Other laboratory tests, such as serum iron and ferritin (sFt) concentrations, total iron-binding capacity (TIBC), and transferrin saturation can be used to distinguish between CHA and ACI, although they can be unreliable in some cases due to their poor specificity and their involvement in the acute phase response [6,8,15]. Newer parameters used in human medicine, such as serum transferrin receptor (sTfR) concentration or the sTfR/log sFt ratio, are currently unavailable in veterinary medicine [13,16]. The only test currently considered as the gold standard of iron status in veterinary medicine is the quantification of total body iron stores, either by liver biopsy (dry matter analysis) or bone marrow cytology/biopsy (iron staining), which are invasive procedures [4,13].

The Sysmex XN-1000V (Sysmex Corporation, Kobe, Japan) analyzer provides advanced hematology parameters, such as the reticulocyte hemoglobin equivalent (RET-He), delta hemoglobin (Delta-He), and the percentage of hypochromic red blood cells (%HYPO-He), which can be useful for the classification of non-regenerative anemias [4,6,7]. RET-He shows the mean hemoglobin concentration in reticulocytes. Since these cells have recently been released from the bone marrow, RET-He acts as a real-time evaluation of iron availability [6,13]. Delta-He represents the difference in hemoglobin concentration between reticulocytes (RET-He) and circulating mature erythrocytes (RBC-He) [6,7]. In human medicine, Delta-He has been proven to differentiate between absolute and functional iron deficiency [7,17]. In order to achieve a better sensitivity and specificity, and also aid in treatment monitoring, a grid graphic (the “Hema-plot”) considering both RET-He and Delta-He is used in humans [7]. Whether a similar approach could be useful in veterinary medicine is unknown. Recent studies in human medicine have also proposed Delta-He as a marker of acute inflammation, even in the absence of anemia, and proved its utility in conditions such as peritonitis or sepsis [7,17]. Whether Delta-He could also fulfill this role in veterinary medicine is currently unknown. Finally, although %HYPO-He (percentage of circulating erythrocytes with a cellular hemoglobin content < 17 pg) is used in humans to detect iron deficiency and monitor iron supplementation [18], its role in the diagnosis of CHA and ACI and its application in veterinary medicine are still unclear.

Since the technology used for the measurement of RET-He, Delta-He, and %HYPO-He varies between analyzers, it is crucial to establish specific reference intervals (RIs) for each equipment [6,10]. To the authors’ knowledge, there are no reports of these parameters using the Sysmex XN-1000V analyzer in dogs or cats.

The objectives of this study were the following: (a) to determine the utility of RET-He, Delta-He, and %HYPO-He in the diagnosis of canine and feline CHA and ACI using the Sysmex XN-1000V; (b) to evaluate the utility of a combined plot using RET-He and Delta-He for the diagnosis of CHA and ACI in these species; (c) to study the variations in Delta-He in dogs and cats with acute inflammatory diseases; and (d) to characterize the RIs of these parameters in healthy dogs and cats using the Sysmex XN-1000V.

## 2. Materials and Methods

### 2.1. Case Selection and Classification

Records from dogs and cats referred to the Veterinary Teaching Hospital of the University of Cordoba between February 2021 and May 2023 were retrospectively selected. Only cases with a complete medical history and a complete blood count (CBC) using the XN-1000V analyzer (Sysmex Corporation, Kobe, Japan) were included in the study. Inclusion and exclusion criteria used in the study are shown in Table 1.

For the first objective, the clinical history, physical examination notes, ancillary diagnostic tests, and laboratory results for dogs and cats with non-regenerative anemia (reticulocyte count (RET) <110 × 10^3^/μL in dogs and <50 × 10^3^/μL in cats) were compiled, and animals were classified as CHA or ACI according to the criteria shown in Table 1. Animals included in the CHA group were those with non-regenerative anemia and a final diagnosis proving chronic bleeding (gastrointestinal ulcerations, marked/severe thrombocytopenia, chronic hematuria without azotemia, internal bleeding, history of trauma or surgery). In the ACI group, animals with non-regenerative anemia and either a final diagnosis (peritonitis, pancreatitis, pneumonia, pyometra, polyarthritis, leishmaniosis, or other systemic infection) or suspicion (leukocytosis, fever) of inflammatory disease were included. Only cases where the available clinical information and diagnostic results were deemed sufficient to allow a definitive classification to one of the groups were included in the study. Any animal with an unclear origin of its anemia was discarded from the study (Table 1).

In order to determine the effect of acute inflammation on RET-He, Delta-He, and %HYPO-He (second objective), data from non-anemic dogs with a recent (less than one week of evolution) onset of fever or leukocytosis or evidence of a recent inflammatory disease (previously mentioned) were selected (Table 1). For the last objective, RIs were calculated using blood samples obtained from healthy patients during an annual health program check. Animals were considered healthy based on a normal physical examination, with a CBC and biochemistry profile (total proteins, albumin, globulins, total bilirubin, urea, creatinine, ALT, and ALP) within the reference ranges.

The following CBC reference ranges (validated in our institution) were used to confirm/discard anemia, leukocytosis, and thrombocytopenia: red blood cell count (RBC) 5–8.6 × 10^6^/μL, hematocrit (HCT) 35–52%, hemoglobin (Hb) 12–20 g/dL, leucocyte count (WBC) 5–17 × 10^3^/μL, and platelet count (PLT) 200–500 × 10^3^/μL for dogs; and RBC 5–12 × 10^6^/μL, HCT 30–45%, Hb 10–15 g/dL, WBC 3–17 × 10^3^/μL, and PLT 250–550 × 10^3^/μL for cats, respectively.

Repeated samples from the same animal were purged from the database. Data corresponding to greyhounds (with breed-specific hematological references), Shar Pei, Chow Chow, Akita, and Shiba Inu (which have been shown to present breed-related microcytosis that can alter reticulocyte variables) were discarded [10].

A total number of 1959 dogs and 595 cats were initially studied. From those, 636 dogs and 277 cats were excluded (Table 1). Thus, a total number of 1323 dogs and 318 cats were finally included. Of these, 969 dogs and 153 cats were considered healthy, while 194 dogs and 82 cats were included in the AID groups (non-anemic animals with some inflammatory disease). Concerning the anemic animals, 61 dogs and 37 cats were included in the CHA groups, whereas 99 dogs and 46 cats were classified as ACI.

The Institutional Animal Care and Use Committee of the Veterinary Teaching Hospital of the University of Cordoba (2021PI/02, date: 8 February 2021) approved this study and owner consent was obtained.

### 2.2. Sample Handling and Measurements

Blood samples were collected by cephalic or jugular venipuncture into K3-EDTA-containing tubes and analyzed using the Sysmex XN-1000V analyzer. The instrument’s graphical information was inspected and the following parameters were retrieved: RBC, HCT, Hb, MCV, mean corpuscular hemoglobin (MCH), MCHC, red cell distribution width (RDW), RET, RET-He, RBC-He, Delta-He, %HYPO-He, WBC, and PLT.

Samples with annotations made by the technicians indicating the presence of macroscopic hemolysis or lipemia were excluded. Internal quality assessments were performed weekly using the manufacturer’s quality control material (XNCHECK Level 1 and Level 2; Sysmex Corporation, Kobe, Japan).

### 2.3. Statistical Analysis

The Kolmogorov–Smirnov test was used to assess normality. Results are expressed as mean ± standard deviation (SD) or median and interquartile range (IQR, 25th–75th percentiles) according to the distribution. Tukey’s hinges test was used to calculate the median and percentiles.

Groups were compared using a one-way ANOVA with Tukey’ post hoc test or the Kruskal–Wallis test with Dunn’s post hoc test, according to normality. Receiver operating characteristic (ROC) curve analysis was performed to determine the sensitivity, specificity, and cut-off values of each parameter. Cut-off values corresponding to RET-He and Delta-He were used to construct a grid graphic.

Reference intervals were calculated using a nonparametric method in dedicated software (Reference Value Advisor v. 2.1. freeware; available at: http://www.biostat.envt.fr/reference-value-advisor/; accessed on 7 September 2024), following the recommendations by the American Society of Veterinary Clinical Pathology [19]. RIs are presented as two-sided 90% confidence intervals (CIs).

Statistical analyses were performed using dedicated software (GraphPad Prism 9, San Diego, CA, USA). Values with *p* < 0.05 were considered significant.

## 3. Results

### 3.1. Conventional Hematological Parameters in Dogs and Cats with AID, CHA, and ACI

In dogs, both anemic groups (CHA and ACI) had significantly (*p* < 0.02) lower MCV, MCH, and MCHC compared to healthy animals (Table 2). PLT was significantly (*p* < 0.03) diminished in dogs with CHA, while WBC was significantly (*p* < 0.005) higher in ACI dogs compared to healthy ones (Table 2). WBC was also significantly (*p* < 0.005) higher in cats with ACI than in healthy ones (Table 2).

When CHA and ACI were compared, no conventional erythrocytic parameter was different between them, neither in dogs nor in cats.

Dogs with AID had significantly (*p* < 0.0001) higher WBC and MCH, while cats with AID showed significantly (*p* = 0.007) higher PLT compared to healthy animals (Table 2). Concerning erythrocytic parameters, the differences between the AID and anemic groups in both species were identical to those mentioned for the control groups (Table 2). Additionally, AID dogs showed significantly higher PLT and WBC (*p* < 0.05) compared to dogs with CHA.

### 3.2. RET-He in Dogs and Cats with AID, CHA, and ACI

**Figure 1:**
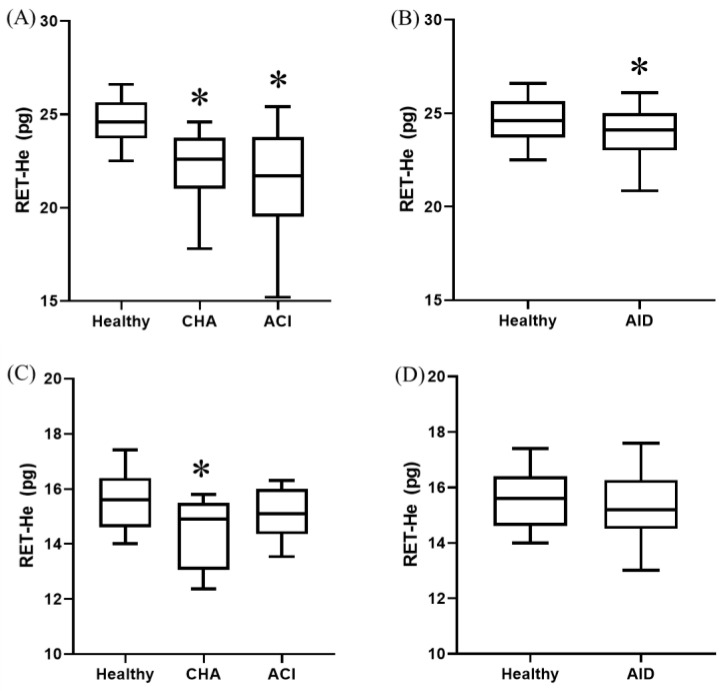
RET-He in different groups of dogs (**A**,**B**) and cats (**C**,**D**). Box and whisker plot. The first and third quartiles form the limits of the box, with the internal line representing the median value. Whiskers correspond to the 10–90% percentile. ACI, anemia of chronic inflammation; AID, acute inflammatory disease; CHA, chronic hemorrhagic anemia. * *p* < 0.05 vs. healthy.

In dogs, both anemic groups showed significantly (*p* < 0.0001) lower RET-He compared to the healthy animals. This finding was only observed in cats with CHA (Figure 1).

No significant differences were observed in RET-He between animals with CHA and ACI, neither in dogs nor in cats.

Dogs with AID presented significantly (*p* < 0.0001) lower RET-He values compared to healthy ones (Figure 1), with no differences seen in cats.

### 3.3. Delta-He in Dogs and Cats with AID, CHA, and ACI

**Figure 2:**
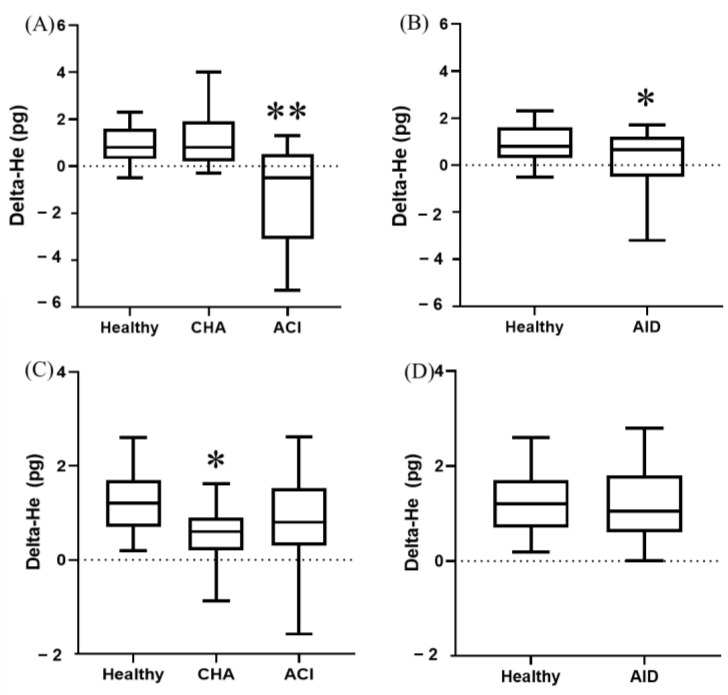
Delta-He in different groups of dogs (**A**,**B**) and cats (**C**,**D**). Box and whisker plot. The first and third quartiles form the limits of the box, with the internal line representing the median value. Whiskers correspond to the 10–90% percentile. ACI, anemia of chronic inflammation; AID, acute inflammatory disease; CHA, chronic hemorrhagic anemia. * *p* < 0.05 vs. healthy. ** *p* < 0.05 vs. every other group.

When anemic groups were compared to healthy animals, Delta-He was significantly lower (*p* < 0.0001) in dogs with ACI and cats with CHA (Figure 2).

In dogs, Delta-He was significantly (*p* < 0.0001) lower in ACI compared to CHA (Figure 2). No significant differences were observed in Delta-He between anemic cats.

Dogs with AID had significantly (*p* < 0.0001) lower Delta-He values compared to healthy ones (Figure 2). No differences were seen in cats.

### 3.4. %Hypo-He in Dogs and Cats with AID, CHA, and ACI

**Figure 3:**
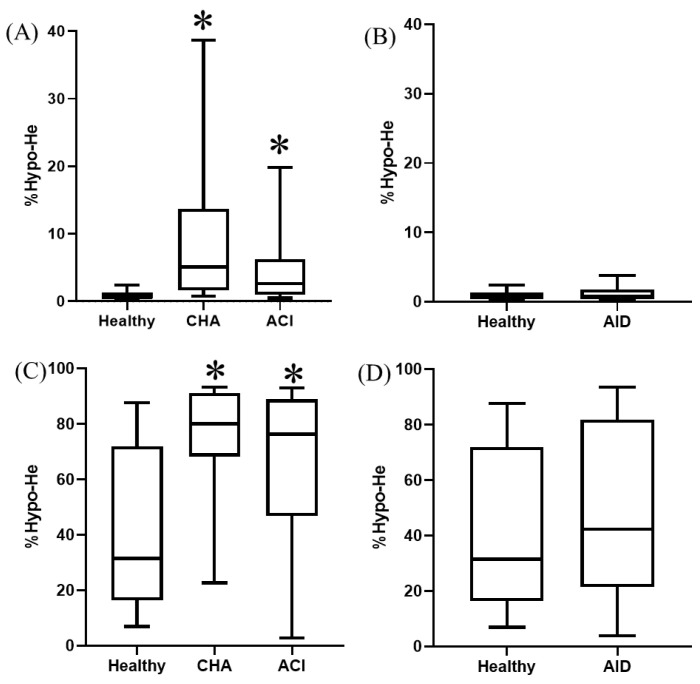
%Hypo-He in different groups of dogs (**A**,**B**) and cats (**C**,**D**). Box and whisker plot. The first and third quartiles form the limits of the box, with the internal line representing the median value. Whiskers correspond to the 10–90% percentile. ACI, anemia of chronic inflammation; AID, acute inflammatory disease; CHA, chronic hemorrhagic anemia. * *p* < 0.05 vs. healthy.

Both anemic groups showed significantly (*p* < 0.0004) higher %Hypo-He compared to healthy animals, both in dogs and cats (Figure 3).

No significant differences were observed in %Hypo-He between animals with CHA and ACI or between healthy and AID animals, neither in dogs nor in cats.

### 3.5. Vet Hema-Plot

**Figure 4:**
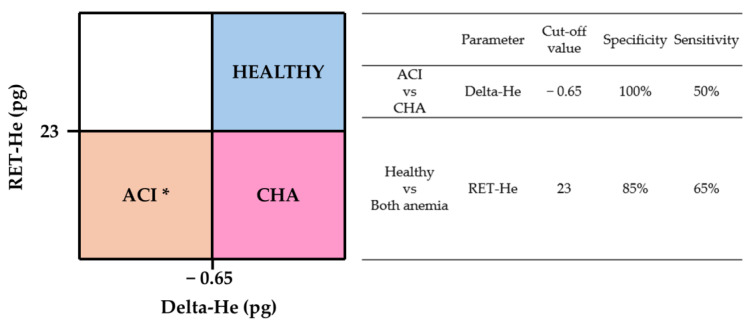
“Vet-Hema plot”. Grid graphic for the recognition of chronic hemorrhagic and chronic inflammatory anemia in dogs using the combination of RET-He and Delta-He in the Sysmex XN-V1000. ACI, anemia of chronic inflammation; CHA, chronic hemorrhagic anemia. * Non-anemic dogs with acute inflammatory diseases (AID) can also be found in this quadrant.

The Vet Hema-plot (Figure 4) was constructed following the same subdivisions and using the same parameters as those displayed in the human Hema-plot version [7]. We chose the cut-off values with higher specificity and sensitivity between each quadrant according to the ROC curves (Figure 4). This plot was only created for dogs due to marked overlapping and poor sensitivity and specificity of cut-off values in cats. ROC curves are compiled in Supplementary Figure S1.

### 3.6. RIs for RET-He, Delta-He, and %Hypo-He in Healthy Dogs and Cats Using the Sysmex XN-1000V

In total, 969 dogs and 153 cats were used to calculate the RIs for RET-He, Delta-He, and %Hypo-He (Table 3). The frequency distributions of these variables are compiled in Supplementary Figures S2 and S3.

## 4. Discussion

While both RET-He and Delta-He were lower in dogs with CHA and ACI compared to healthy ones, only Delta-He was significantly different between both types of anemia. Neither %Hypo-He nor conventional hematological parameters were useful to separate these two conditions. In addition, Delta-He was also significantly low in dogs with AID, confirming its value as an inflammatory marker, similar to humans [7,20]. In cats, these parameters were not significantly different between CHA and ACI cases. Thus, RET-He and Delta-He in the Sysmex XN-1000V, in conjunction with other clinical and laboratory findings, can help clinicians to find the cause of non-regenerative anemia in dogs.

This is the first study concerning the use of RET-He and Delta-He in veterinary medicine using the Sysmex XN-1000V. Previous reports have been performed with different analyzers or have measured CHr (similar to RET-He) and CH-delta (equivalent to Delta-He) [4,6,8,10]. The correlations between these parameters in different analyzers are high (r^2^ = 0.92), and findings are usually extrapolatable between them [8,14,21].

Our first objective was to evaluate the utility of these advanced hematological parameters in the diagnosis of chronic hemorrhage anemia (CHA) and anemia of chronic inflammation (ACI). Routinary hematologic variables were unable to distinguish between these conditions, with both groups showing a similar degree of non-regenerative anemia (low RBC, HCT, Hb) in dogs and cats. Additionally, in anemic dogs, both groups showed low MCV, MCH, and MCHC. Since microcytosis and/or hypochromia have been reported in dogs with a variety of chronic diseases due to a prolonged sequestration of iron [4,5,10], these findings are not currently recognized as pathognomonic for CHA. Moreover, microcytosis can be found in certain Asian dog breeds or in other conditions such as portosystemic shunts (PSS) [4,10]. In our study, neither patients from these breeds nor dogs with a diagnosis of PSS (or compatible laboratory anomalies) were included.

Previous studies have described low RET-He in canine primary or absolute iron deficiency [4,13]. However, newer reports have demonstrated that low RET-He values also appear in dogs with inflammatory conditions [4,10,11]. Our findings are in accordance with these latter works, with both CHA and ACI dogs displaying low RET-He, without significant differences between groups. Thus, RET-He alone should not be used to differentiate between CHA and ACI in dogs.

While low RET-He values have been observed in cats with any cause of iron-limited erythropoiesis [14,22,23], no studies have compared CHA and ACI in this species. In our study, both groups show low RET-He, but this difference was only significant between CHA and healthy cats. Moreover, no differences were found between CHA and ACI. Thus, although low RET-He appears to be more specific for CHA in cats, this parameter alone is not reliable in determining the cause of anemia in this species. Low RET-He values have also been observed in cats with chronic kidney disease (CKD) [15]. In our study, any animal with a diagnosis of CKD was excluded to avoid the confounding effects of renal failure on hematopoiesis.

Delta-He is calculated based on RET-He and RBC-He (Delta-He = RET-He–RBC-He). In humans with absolute iron deficiency, due to the long-lasting absence of iron, both RET-He and RBC-He are diminished, resulting in an unchanged or slightly low Delta-He [7,17]. In contrast, patients with inflammatory conditions show low Delta-He values due to hepcidin primarily impairing the hemoglobinization of reticulocytes (thus causing a more profound decrease in RET-He compared to RBC-He) [7,24]. In our study, Delta-He was significantly different between dogs with CHA and ACI. On the other hand, a previous report using a smaller cohort of dogs with both conditions (*n* = 27 and 35, respectively) and a different analyzer found overlapping values between the groups [10]. Our results mimic those described in humans [7,17,24], underlining the utility of this parameter in this species.

In relation to cats, and to the authors’ knowledge, this is the first report of Delta-He in this species. Contrary to dogs, Delta-He values were only significantly low in CHA animals compared to healthy ones (similarly to RET-He). This finding underlines the dissimilarity in hemoglobinization between the studied species, which could be related to differences in the effects of hepcidin or in the bone marrow iron storage between them [15].

Delta-He has recently gained wide interest in human medicine as a marker of inflammation. Several studies have found low Delta-He in patients with peritonitis and sepsis, described its prognostic value in these conditions, and underlined its ability to detect the resolution of the disease [7,17,25]. Low Delta-He in these disturbances is linked to low RET-He values, most probably due to hepcidin acting as an acute phase reactant [7]. Our results in dogs are similar to these studies, with canine AID showing significantly lower Delta-He and RET-He compared to healthy animals. These findings are also in line with a previous report that demonstrated lower RET-He values in dogs with inflammation and high C-reactive protein (CRP) compared to animals without inflammation [26]. On the other hand, no differences were observed in cats, which could be related to idiosyncrasies in hepcidin or hemoglobinization in this species.

%HYPO-He represents the percentage of circulating erythrocytes with a cellular hemoglobin content < 17 pg, a cut-off value proposed in human medicine [6,18]. This parameter is an early indicator of iron-deficient erythropoiesis in humans, preceding the presence of microcytic erythrocytes in circulation [27]. It is used to recognize iron deficiency in blood donors and monitor iron supplementation [6,27], as it is not influenced by infection or inflammation [27,28]. No studies evaluating the clinical utility of %HYPO-He have been published in veterinary medicine. Our results, both in dogs and cats, concur with those reported in humans, with both anemic groups showing significantly higher %HYPO-He than healthy animals, although no differences between CHA and ACI were seen.

Combinations of several hematological variables are commonly used in human medicine for the diagnosis of iron-dependent anemias [7,20,27]. Of those considering RET-He and Delta-He, the Hema-plot is one of the most useful, both for the diagnosis and for the treatment monitoring of these conditions [7]. We used our data to construct a graphic grid following the structure of the Hema-plot, which we have called the Vet Hema-plot. According to our results, this approach could be useful in anemic dogs to distinguish between CHA (low RET-He with normal Delta-He) and ACI (low RET-He and low Delta-He). In the Vet Hema-plot, AID dogs also show low RET-He and low Delta-He (similar to ACI). However, since AID dogs are non-anemic, this could have been detected using conventional CBC results. It is important to note that, due to the high degree of overlapping values in our study, the cut-off values between each quadrant should be used with caution and the Vet Hema-plot should be interpreted along with additional information (anamnesis, physical exam, CBC, and ancillary laboratory testing). We did not construct a feline version due to the marked overlap between cut-off values.

Our last objective was focused on establishing RIs for these parameters using the Sysmex XN-1000V on healthy animals. Although there is a good correlation among analyzers [8,14,21], due to differences in the technology used to measure these parameters, the extrapolation of RIs is not advised. Compared to previous reports using different analyzers, our RIs for feline RET-He were similar [14], while the lower limits of our canine RET-He and Delta-He RIs were less high [4,6,8,13,26]. RIs for canine %Hypo-He were wider in our data compared to the results from a previous study [6]. The feline %Hypo-He RI was wide, and many healthy cats showed very high percentages of erythrocytes classified as hypochromic. This finding could be related to species-specific differences in hemoglobin content in normal erythrocytes and the pre-established human cut-off value used to recognize these cells not being suitable for cats.

The present study has some limitations. First, we did not measure specific biochemical parameters such as serum iron or ferritin concentrations, TIBC, or transferrin saturation to determine the iron status of our patients, relying on clinical signs and ancillary results to differentiate between CHA and ACI. While several previous studies have used a similar methodology [4,8,10], and these iron-related tests could show overlap in some cases [6,8,15], they could have strengthened our results. In a similar way, data on the concentration of acute phase proteins or other mediators (hepcidin, interleukins, etc.) could have been helpful for the unambiguous recognition of animals in the inflammatory groups. Consequently, we cannot categorically claim that RET-He and Delta-He can be used to differentiate between every case of canine absolute and functional iron deficiency, since we have only studied cases with chronic hemorrhagic and chronic inflammatory anemias. Second, we cannot absolutely dismiss that the anemia in some animals could have been multifactorial, although any animal with an unclear classification was excluded. Finally, animals were considered healthy based on normal clinical history, physical examination, and a CBC and biochemistry results within reference ranges, but subclinical diseases or infections (such as FIV or FeLV in cats) could have altered some of the results.

## 5. Conclusions

The combined use of Delta-He and RET-He in the Sysmex XN-1000V can help to identify AID, CHA, and ACI in dogs. These parameters are an informative, rapid, and non-invasive tool that can assist clinicians to classify the type of anemia in these animals. These parameters should always be interpreted in conjunction with clinical signs and additional laboratory findings. Further research on new applications of these innovative parameters in veterinary medicine (prognostic value, therapy monitoring) could be compelling.

## Figures and Tables

**Table 1:** Inclusion and exclusion criteria for dogs and cats of the study. The number of animals excluded by each criterion is shown between parentheses.

Group	Inclusion Criteria	Exclusion Criteria (Dogs//Cats)
Healthy	-Normal anamnesis and physical examination. -CBC and biochemistry panel within reference ranges.	-Delayed CBC after blood collection (>6 h) (151 dogs//101 cats). -Marked lipemia and/or hemolysis (35 dogs//10 cats). -Previous blood transfusion (<30 days) (17 dogs//1 cat). -Younger than 3 months (45 dogs//4 cats). -Greyhound, Shar Pei, Chow Chow, Akita and Shiba Inu (79 dogs//0 cats).
AID	-Evidence (gastroenteritis, peritonitis) or suspicion (fever, leukocytosis) of an acute (<1 week of evolution) inflammatory disease.	-All of those mentioned in the healthy group (327 dogs//116 cats). -Anemia (of any type). -Leukopenia (10 dogs//2 cats).
CHA	-Non-regenerative anemia with evidence of chronic blood loss (diagnosis of anemia due to gastrointestinal hemorrhage, thrombocytopenia, trauma, surgery, chronic hematuria, or internal bleeding).	-All of those mentioned in the healthy group (327 dogs//116 cats). -Doubtful classification between groups (209 dogs//91 cats). -Evidence of renal disease (41 dogs//34 cats). -Evidence or suspicion (concurrent leukopenia) of bone marrow failure (20 dogs//22 cats). -Evidence of hemolysis (29 dogs//12 cats).
ACI	-Non-regenerative anemia with evidence (peritonitis, pancreatitis, pneumonia, systemic infection) or suspicion (fever, leukocytosis) of a chronic inflammatory disease.	

ACI, anemia of chronic inflammation; AID, acute inflammatory disease; CHA, chronic hemorrhagic anemia.

**Table 2:** Hematological results for different groups of animals using the Sysmex XN-1000V.

	Dogs (*n* = 1323)				Cats (*n* = 318)			
	Healthy (*n* = 969)	AID (*n* = 194)	CHA (*n* = 61)	ACI (*n* = 99)	Healthy (*n* = 153)	AID (*n* = 82)	CHA (*n* = 37)	ACI (*n* = 46)
HCT (%) ^a^	45.8 ± 5.5	46.5 ± 6.3	26.1 ± 5.8 ^1,2^	26.0 ± 6.4 ^1,2^	36.5 ± 5.1	36.7 ± 5.6	20.5 ± 5.4 ^1,2^	23.7 ± 9.1 ^1,2^
RBC (10^6^/µL) ^b^	6.6 (1.2)	6.3 (1.5)	4.3 (1.2) ^1,2^	4.1 (1.7) ^1,2^	8.2 ± 1.3	8.3 ± 1.4	4.7 ± 1.2 ^1,2^	5.4 ± 2.3 ^1,2^
Hb (g/dL)	15.9 (3.0)	15.5 (3.9)	8.7 (3.0) ^1,2^	9.0 (3.6) ^1,2^	11.9 (2.6)	12.2 (3.0)	7.0 (3.2) ^1,2^	8.0 (2.2) ^1,2^
MCV (fL) ^b^	69.7 (3.8)	70.1 (4.8)	67.6 (3.6) ^1,2^	68.5 (5.3) ^1,2^	44.7 ± 3.7	44.8 ± 3.8	43.7 ± 4.0	44.3 ± 5.2
MCH (pg)	24.1 (1.4)	24.2 (2.0) ^1^	22.2 (3.1) ^1,2^	22.8 (2.3) ^1,2^	14.7 (1.9)	14.9 (1.7)	14.2 (1.8)	14.4 (1.7)
MCHC (g/dL) ^a^	34.6 ± 1.1	34.7 ± 1.9	33.5 ± 3.7 ^1,2^	33.0 ± 1.9 ^1,2^	33.1 ± 1.6	33.3 ± 1.9	32.7 ± 1.8	33.9 ± 6.2
RDW (fL) ^b^	34.2 (3.5)	34.6 (4.4)	34.4 (8.4)	34.0 (6.7)	34.4 ± 5.4	35.9 ± 6.6	35.7 ± 6.6	35.4 ± 6.4
RET-He (pg)	24.6 (1.9)	24.1 (2.0) ^1^	22.6 (2.7) ^1,2^	21.7 (4.3) ^1,2^	15.6 (1.8)	15.2 (1.9)	14.9 (2.4) ^1,2^	15.1 (1.6)
RBC-He (pg)	23.7 (1.3)	23.7 (1.6)	21.6 (2.9) ^1,2^	22.9 (2.6) ^1,2,3^	14.2 (1.5)	13.9 (1.8)	14.0 (2.3)	14.1 (1.7)
Delta-He (pg)	0.80 (1.3)	0.65 (1.7) ^1^	0.80 (1.7) ^2^	−0.50 (3.6) ^1,2,3^	1.20 (1.0)	1.05 (1.2)	0.60 (0.7) ^1,2^	0.80 (1.2)
%Hypo-He (%)	0.60 (0.9)	0.75 (1.4)	5.10 (12) ^1,2^	2.60 (5.2) ^1,2^	31.5 (55)	42.3 (60)	80.1 (23) ^1,2^	76.3 (42) ^1^
PLT (10^3^/µL)	243 (139)	249 (192)	171 (242) ^1,2^	218 (204) ^2^	257 (109)	341 (314) ^1^	209 (167)	215 (104)
WBC (10^3^/µL)	9.2 (4.3)	20.7 (9.3) ^1^	9.8 (7.7) ^2^	24.5 (10) ^1,3^	9.1 (4.5)	17.4 (19)	10.5 (4.4)	12.8 (3.6) ^1^

Data are expressed as median (IQR, interquartile range) or mean ± standard deviation (SD), according to distribution. ACI, anemia of chronic inflammation; AID, acute inflammatory disease; CHA, chronic hemorrhagic anemia; Delta-He, delta hemoglobin; Hb, hemoglobin concentration; HCT, hematocrit; MCH, mean corpuscular hemoglobin; MCHC, mean corpuscular hemoglobin concentration; MCV, mean corpuscular volume; PLT, platelet count; RBC, red blood cell count; RBC-He, mature erythrocyte hemoglobin equivalent; RDW, red blood cell distribution width; RET-He, reticulocyte hemoglobin equivalent; WBC, leucocyte count; %Hypo-He, percentage of hypochromic erythrocytes. ^1^ *p* < 0.05 vs. healthy animals. ^2^ *p* < 0.05 vs. AID. ^3^ *p* < 0.05 vs. CHA. ^a^ Parameter with normal distribution both in dogs and cats. ^b^ Parameter with normal distribution in cats.

**Table 3:** Reference intervals for RET-He, Delta-He, and %Hypo-He in dogs and cats using the Sysmex XN-1000V.

	Parameter	Lower Limit	Upper Limit	CI 90% of Lower Limit	CI 90% of Upper Limit
Dogs (*n* = 969)	RET-He (pg)	21.1	27.2	20.7–21.3	27.1–27.4
	Delta-He (pg)	−1.7	3.3	−2.0–−1.5	3.1–3.5
	%Hypo-He (%)	0.2	5.1	0.2–0.2	4.7–5.7
Cats (*n* = 153)	RET-He	13.7	19.3	13.7–13.7	18.4–22.8
	Delta-He	−0.8	4.0	−0.9–−0.2	3.2–5.3
	%Hypo	3.5	94.1	0.7–4.2	90.7–96.1

CI, confidence interval; Delta-He, delta hemoglobin; RET-He, reticulocyte hemoglobin equivalent; %Hypo-He, percentage of hypochromic erythrocytes.

## Data Availability

The data presented in this study are available upon request to the corresponding author.

## Supplementary Materials

The following supporting information can be downloaded at: https://www.mdpi.com/article/10.3390/ani14223215/s1, Figure S1: Receiver operating characteristic curve of (**A**) Delta-He for the differential diagnosis between CHA and ACI in dogs; and (**B**) RET-He for the differential diagnosis between healthy dogs and both anemic groups. Figure S2: Frequency distributions and reference intervals for RET-He, Delta-He, and %Hypo-He in dogs; Figure S3: Frequency distributions and reference intervals for RET-He, Delta-He, and %Hypo-He in cats.
