# Supplementary material for: Using RET-He and Delta-He in the Sysmex XN-1000V Analyzer to Differentiate Between Chronic Hemorrhagic and Chronic Inflammatory Anemia in Small Animals
Source: Animals (Basel). 2024 Nov 9;14(22):3215. doi: 10.3390/ani14223215 (PMC11591344; doi:10.3390/ani14223215)
Supplement: Supplementary file 1 [file animals-14-03215-s001.zip › Supplementary Figure S1.pdf]

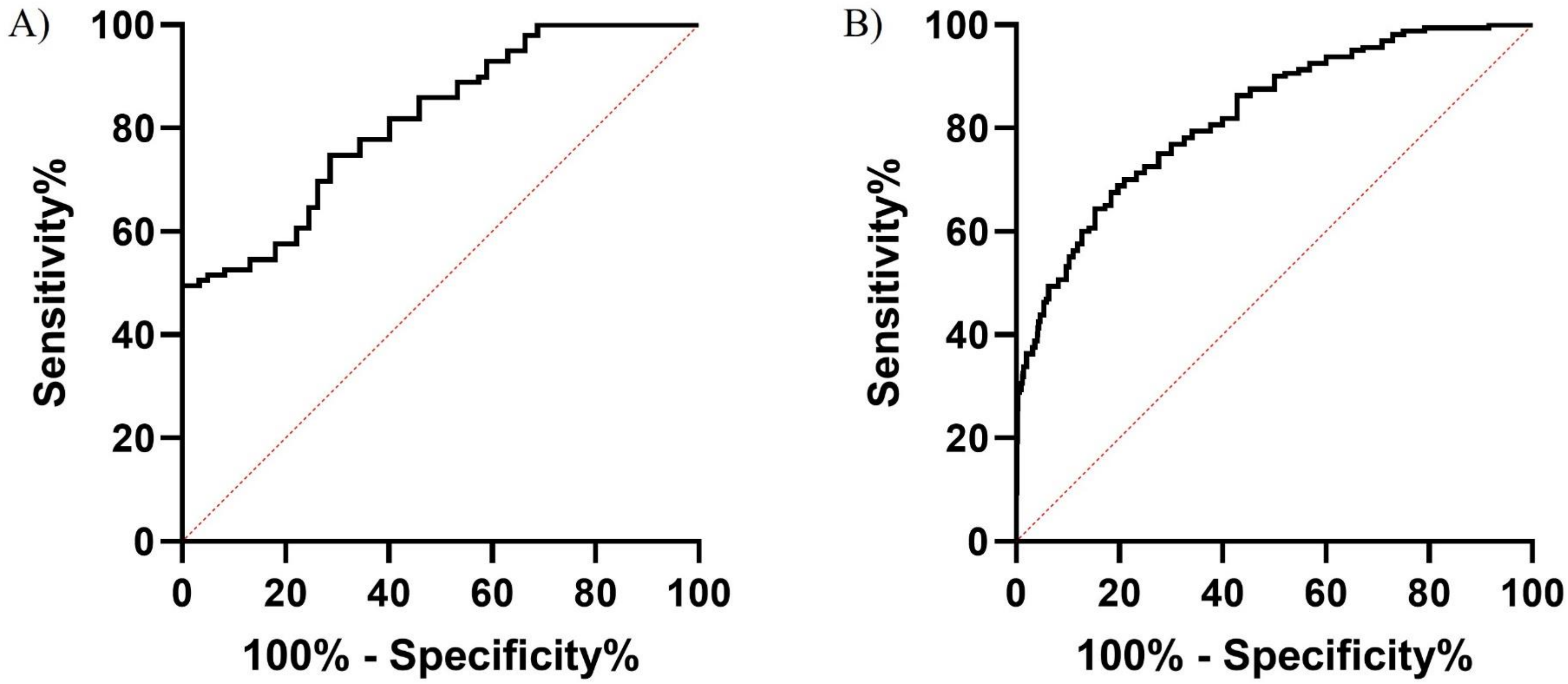

Supplementary Figure S1. Receiver operating characteristic curve of (A) Delta-He for the differential diagnosis between CHA and ACI in dogs; and (B) RET-He for the differential diagnosis between healthy dogs and both anemic groups.
