# Supplementary material for: Using RET-He and Delta-He in the Sysmex XN-1000V Analyzer to Differentiate Between Chronic Hemorrhagic and Chronic Inflammatory Anemia in Small Animals
Source: Animals (Basel). 2024 Nov 9;14(22):3215. doi: 10.3390/ani14223215 (PMC11591344; doi:10.3390/ani14223215)
Supplement: Supplementary file 1 [file animals-14-03215-s001.zip › Supplementary Figure S2.pdf]

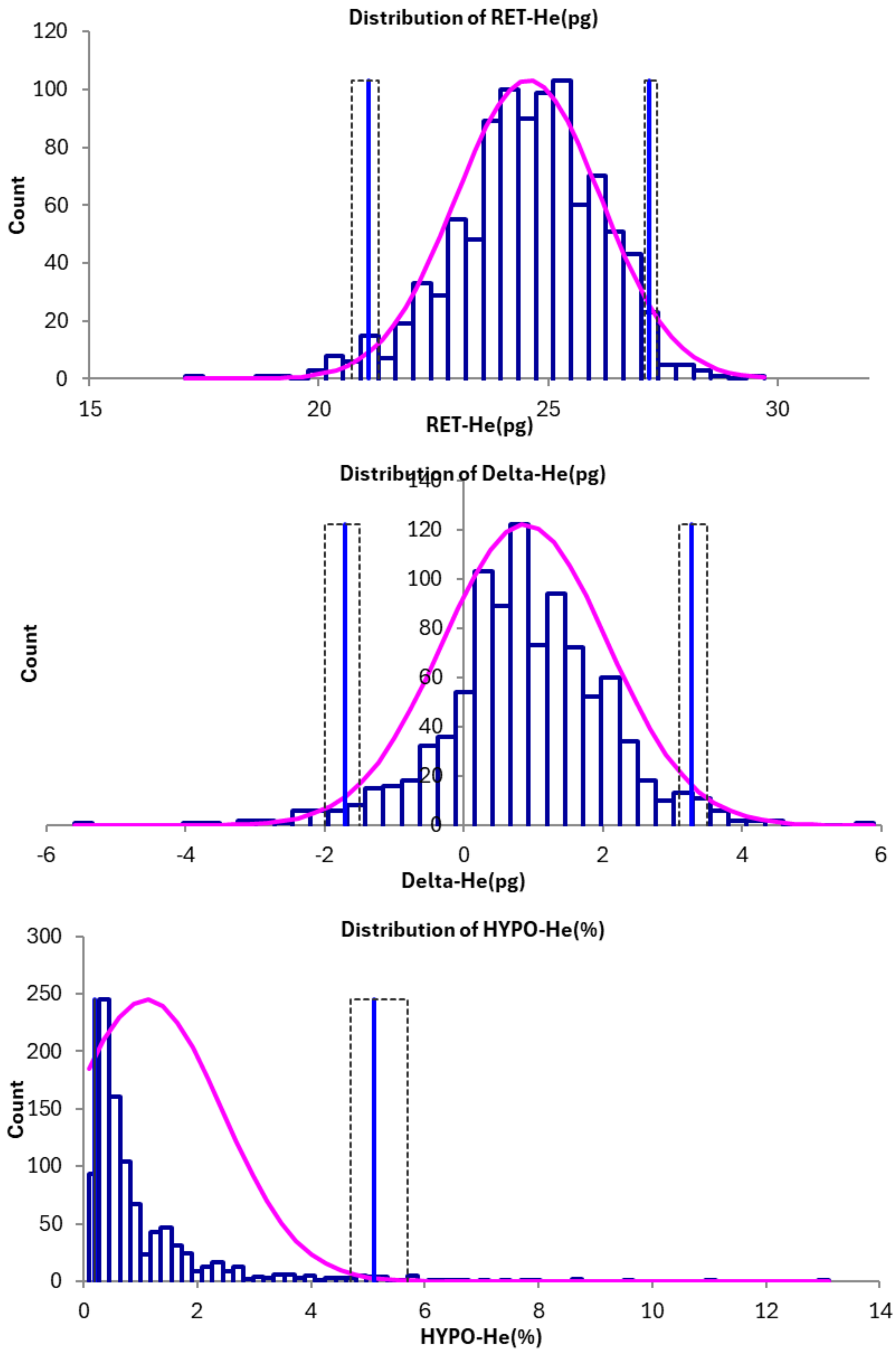

Supplementary Figure S2. Frequency distributions and reference intervals for RET-He, Delta-He and %Hypo-He in healthy dogs. The observed distribution is represented by the vertical black columns, while the red curve is the fitted distribution. Reference limits are drawn as vertical blue lines. Dotted bar surrounding those limits are the 90% confidence intervals.
